# From crisis to commitment: nursing students’ sense of belonging during and immediately after the covid-19 pandemic

**DOI:** 10.1080/17482631.2026.2663572

**Published:** 2026-04-24

**Authors:** Reem Saeed Alghamdi, Monirah Albloushi, Badr Ayed Alenazy, Hilah Almater, Nasima Awaji, Reem Fallatah, Amal Barasheed, Gada Albothi

**Affiliations:** a King Saud University, College of Nursing, Riyadh, Saudi Arabia; b Nursing Administration, Northern Bordars Health Cluster, Saudi Arabia

**Keywords:** Nursing students, professional identity, resilience, sense of belonging, career choice

## Abstract

**Background:**

Following the COVID-19 pandemic, global health systems continue to face strain. Understanding how crises shape professional development is vital for strengthening nursing education and sustaining the workforce. This study explored how nursing students in the Kingdom of Saudi Arabia maintained their sense of belonging to the nursing profession after the pandemic and how these experiences affected their career decisions.

**Methods:**

An exploratory, descriptive, and qualitative design was adopted using semi-structured interviews. Using purposive sampling, eight undergraduate and postgraduate nursing students from various Saudi universities participated in semi-structured interviews conducted via Zoom. Data were analyzed using thematic analysis.

**Results:**

Three themes and five subthemes were identified from the participants’ experiences. The themes included social recognition and appreciation, psychological impact of the pandemic, professional identity, and career orientation. These themes were interrelated and encompassed the social, emotional, and professional dimensions of the participants’ experiences. These dimensions often overlap, illustrating how the COVID-19 pandemic has shaped students’ sense of belonging, emotional strength, and commitment to nursing.

**Conclusion:**

The COVID-19 pandemic had a transformative impact on Saudi nursing students’ professional identities, resilience, and sense of belonging. Despite disruptions, students demonstrated adaptability, ethical commitment, and a broadened view of nursing as a socially impactful and multifaceted profession. Thus, incorporating structured mentorship, emotional support, and inclusive learning environments in nursing education, which foster professional identity during and after the easing of pandemic restrictions and other crisis conditions is crucial. Future research should investigate these dynamics across a broader range of institutional and cultural contexts to develop resilient and inclusive nursing education models.

## Introduction

The COVID-19 pandemic has profoundly influenced nursing students’ perceptions of their professional identities, subsequent career decisions, and sense of belonging (Rodríguez-Pérez et al., 2022). Professional identity refers to the internalisation of nursing values, roles, and ethics, whereas a sense of belonging reflects the relational and institutional integration that sustains this identity during training (Kristoffersen, 2021; Levett-Jones & Lathlean, 2008). Professional identity in nursing is strongly influenced by educational experiences, clinical exposure, and societal recognition (Kristoffersen, 2021). The global health crisis significantly shaped the experiences of nursing students worldwide. While the pandemic increased public visibility and recognition of the nursing profession, it also disrupted educational pathways, clinical placements, and students’ psychosocial well-being (Batmaz et al., 2022). This visibility fosters pride and deeper alignment with nursing values, strengthening their identification with the profession (Jackson et al., 2020; Jeon et al., 2020). Numerous studies report that students have witnessed the pivotal role of nurses in patient care, leadership, and public health during the pandemic, which has intensified their sense of belonging to the profession and reinforced their decision to pursue nursing as a lifelong career (Dos Santos, 2020; Head et al., 2022; Kobayashi et al., 2023).

The theoretical underpinnings of a sense of belonging are rooted in psychological and sociological models, reflecting an evolving concept shaped by individuals’ interactions within multiple social systems. Maslow (1943) hierarchy of needs identifies belongingness as a core human need, situated above physiological and safety needs and foundational to self-esteem and self-actualisation. Maslow’s framework makes it clear that belonging is not a static state; it develops through ongoing relationships, acceptance, and integration across family, peer groups, cultural affiliations, workplaces, and geographic communities highlighting its relevance well beyond mere professional status (Maslow, 1943).

Contemporary research increasingly conceptualises belonging as an ongoing, dynamic process involving continuous interactions and relationships, rather than as a singular event or fixed condition. It encompasses subjective feelings of acceptance, integration, and mattering, extending across diverse settings such as family, educational environments, professional teams, and cultural communities not limited to institutional affiliation. In educational contexts, Tinto (1993) model of student retention emphasises the importance of both academic and social integration in fostering a sense of belonging, which, in turn, promotes student persistence. These frameworks offer valuable insights into the mechanisms by which belongingness influences the academic and professional paths of nursing students (Maslow, 1943; Tinto, 1993).

A sense of belonging has been consistently associated with better psychosocial well-being, lower levels of anxiety and depression, and stronger social functioning (Abdulhamed et al., 2024; Allen et al., 2021). In healthcare settings, the ability to feel connected and valued by colleagues and the institution contributes not only to emotional health but also to safer work practices and better patient care (Hall et al., 2024). Transformative Learning Theory (Mezirow, 1991) has been applied in nursing and health education to explain how critical reflection, social discourse, and identity shifts facilitate deeper professional belonging and growth (Pan et al., 2025; Tsimane & Downing, 2020).

A strong sense of belonging has long been recognised as vital to nursing students’ academic success and identity formation (Patel et al., 2024; Singer et al., 2022; Squire et al., 2024). It facilitates engagement, enhances resilience (Christina Dempsey & Barbara, 2016), and fosters a deeper connection with the nursing profession (Squire et al., 2024). For nursing students, belonging is not merely a passive state, but a dynamic and evolving experience shaped by feeling accepted, respected, and included within academic and clinical environments (Albloushi et al., 2019; Levett-Jones & Lathlean, 2008). This reflects a meaningful connection between students and the nursing community, contributing to their professional identity, motivation, and overall educational engagement (Thomas et al., 2023). Students who perceive themselves as integral members of the nursing community are more likely to develop professional values and commitment to the profession (Miao et al., 2024). During the pandemic, many traditional sources of belonging, such as peer support, clinical mentorship, and face-to-face learning, were significantly disrupted. Students commonly experienced isolation, anxiety, and detachment from their academic and clinical environments (Albloushi et al., 2024; Aslan & Pekince, 2021). Clinical placements, which are essential for fostering a sense of professional belonging, were suspended or limited in many institutions during the pandemic. Students were often excluded from healthcare teams because of infection control concerns and the prioritisation of limited resources, leading some to question their place within the nursing community (Hayter & Jackson, 2020). For some students, the pandemic served as a transformative educational experience. Those who were able to volunteer in healthcare settings or observe the dedication and bravery of frontline nurses developed a deeper understanding of the nursing role and a renewed sense of purpose (Raustøl & Tveit, 2023). Exposure to real-world healthcare crises strengthened students’ emotional resilience, ethical grounding, and sense of calling in their profession (Goni-Fuste et al., 2025; Ulenaers et al., 2021). For some students, these crisis experiences acted as turning points in their education, deepening their understanding of nursing as a profession rooted in compassion, advocacy, and public service (Deliktas Demirci et al., 2021). Recent qualitative research has also highlighted the complex and multifaceted experiences of nursing students during the COVID-19 pandemic. Findings from a large multisite qualitative investigation conducted across the United Kingdom revealed that nursing students experienced both professional growth and significant emotional and educational challenges during the pandemic (Brett et al., 2024; Henshall et al., 2023). Students described feelings of pride in contributing to healthcare services and reported strengthened professional identity and resilience; however, they also experienced anxiety, uncertainty, and concerns about their clinical preparedness due to disrupted placements and rapidly changing learning environments. These findings illustrate that the pandemic shaped nursing students’ professional identity and preparedness for practice in complex ways, reflecting both opportunities for professional development and significant educational disruption.

In response to the urgent needs of healthcare systems worldwide, nursing students in several countries volunteered to join the workforce despite the highly stressful and demanding conditions. These volunteer experiences demonstrated students’ resilience and fostered a stronger sense of belonging to the nursing profession, thereby reinforcing a sense of purpose and professional connection (Goni-Fuste et al., 2025; Labrague & de Los Santos, 2021; Li et al., 2023; Martin-Delgado et al., 2021). In the context of the United Arab Emirates, Rosita et al., (2023) have conducted a large-scale cross-sectional study examining how the COVID-19 pandemic influenced nursing students’ attitudes toward their career choices and nursing’s public image. The findings revealed a dominant “no turning back” sentiment among participants, who expressed pride and satisfaction in their professional choice. Crucially, students perceived the heightened public and government recognition of nurses during the pandemic as a powerful affirmation of the value of their role. (Alotaibi et al., 2023) have reported that Saudi nursing students maintained positive attitudes toward the nursing profession during the pandemic. This is particularly significant given the historical context in Saudi Arabia, where nursing has long faced challenges related to public perception and is often viewed as a low-status, subordinate, and culturally inappropriate profession for women (Al-Khunizi et al., 2021; Elmorshedy et al., 2020). The pandemic has served as a turning point in elevating the societal value of nurses and enhancing respect for their profession. In both studies, this increased recognition positively influenced students’ outlook, reinforcing their career commitment and professional identity, and reframing nursing as a respected and vital role in the Gulf region.

According to Jackson et al. (2020), the visibility of nurses’ frontline roles during the COVID-19 pandemic, which were often framed as heroic, inspired many students to reaffirm their commitment to clinical practice. Witnessing the critical role of nurses in managing public health crises reinforces the intrinsic value of the profession and strengthens their motivation to pursue bedside and acute care roles after graduation (Smith et al., 2023). However, the extreme working conditions faced by nurses, including reports of burnout, mental health deterioration, and inadequate institutional support, have led some nursing students to question their long-term future in the profession. Fear of infection, concern for family safety, and moral distress from witnessing patient suffering are frequently cited as reasons for reconsidering nursing as a career (Huang et al., 2020; Labrague & de Los Santos, 2021). For some, the pandemic has sparked interest in alternative nursing pathways, such as public health, education, and administration, which are perceived as less physically and emotionally demanding (LoGiudice & Bartos, 2021). These studies provide valuable insights into cognitive and attitudinal shifts during and after the COVID-19 pandemic. While a growing body of research has examined nursing students’ experiences during this period, much of the literature relies on quantitative designs, with fewer studies offering in-depth qualitative exploration of students’ lived experiences and evolving sense of belonging following the pandemic. A qualitative enquiry is necessary to understand how these crises influence long-term career choices, inform educational reforms, and support the retention of committed and resilient nurses. This study explored how nursing students’ sense of belonging and career choices have evolved following the COVID-19 pandemic, drawing on in-depth experiences to uncover the emotional, relational, and vocational aspects of students’ journeys.

## Methods

### Study design

This study adopted a qualitative exploratory descriptive design to explore how nursing students reflected on their sense of belonging to the nursing profession during the later stages of the COVID-19 pandemic and the immediate period that followed. This design is particularly well-suited for research areas where limited prior knowledge exists, as it allows for an in-depth exploration of the participants’ perspectives (Sandelowski, 2000). The exploratory descriptive approach emphasises the participants’ experiences, facilitating the development of a rich and detailed understanding of the phenomenon. This flexible yet rigorous approach enabled the researchers to remain close to the data and represent the participants’ voices with authenticity and clarity.

### Participants

A purposive sampling strategy was used to recruit nursing students from undergraduate and postgraduate programmes at Saudi universities. These students were actively engaged in both academic coursework and clinical placements, forming the core population involved in developing professional knowledge, skills, and identity. Purposive sampling is commonly used in qualitative research to select individuals with rich and relevant experience related to the phenomenon under study. The final sample included five undergraduate students enroled in the Bachelor of Science in Nursing (BSN) programme, which is a pre‑licensure programme, and three postgraduate students enroled in the Master of Science in Nursing (MSN) programme. The students were recruited from three universities across Saudi Arabia and undertook clinical training in both tertiary and local hospital settings. We employed homogeneous purposive sampling, treating BSN and MSN students as a single group based on their shared identity as nursing students, aligning with the study’s focus on belonging and engagement. Differences in clinical experience and registration were noted as context but not central to the analysis. Including both groups was intended to capture perspectives from different stages of professional education, thereby adding depth to understanding how belonging is perceived within academic contexts. Although no formal data on participants’ years of professional nursing experience were collected, it is acknowledged that graduate students may have entered the programme with prior clinical or professional exposure, which could shape their perspectives differently from those of undergraduate pre‑licensure students. The seventh interview achieved data saturation, and no new themes emerged in the subsequent eighth interview. This sample size aligns with qualitative research guidelines that emphasise the value of small, information-rich samples for generating an in-depth understanding of the phenomenon (Creswell, 2019; Patton, 2014; Polit & Beck, 2017). The inclusion criteria were: (1) current enrolment in a BSN or MSN programme at the time of data collection, and (2) willingness to share experiences related to nursing during the COVID-19 pandemic. Students who had withdrawn or taken a leave of absence from their nursing programme were excluded.

### Data collection

Recruitment was facilitated via email invitations and social media. Interested participants were provided with detailed information about the study's purpose, procedures, and ethical considerations. The study employed purposive sampling with the explicit aim of capturing variation in both academic level and gender. During recruitment, invitations were disseminated to both undergraduate and postgraduate cohorts, and efforts were made to invite and include students identifying as male and female. The recruitment process and participant demographics were reviewed throughout data collection to ensure that both academic levels and genders were represented, thereby achieving a diverse sample in line with the study’s aims. Data were collected between May and September 2022, a period when most COVID-19 restrictions had been lifted in Saudi Arabia and healthcare services were transitioning from the acute crisis phase to routine operations. The study included both undergraduate (BSN) and postgraduate (MSN) nursing students. During the pandemic period, BSN participants were primarily engaged in academic learning and were largely not exposed to clinical placements due to institutional restrictions, which may have influenced their perceptions of professional belonging. In contrast, several MSN participants were practicing nurses who continued working in clinical settings, including COVID-19 units. As a result, the findings reflect both educational experiences and frontline clinical perspectives shaped by the pandemic context. Participants were recruited from three Saudi universities through social media (Twitter, WhatsApp), targeted emails, and flyers distributed via programme offices. A study poster outlined the purpose, procedures, participants’ rights, and contact details, and interested students arranged Zoom interviews directly, ensuring convenience, geographic flexibility, and adherence to post-pandemic safety measures. The researchers were not involved in the participants’ coursework or teaching and had no prior relationships with them.

The interview guide was informed by existing literature. It consisted of open-ended questions designed to explore students’ sense of belonging, their experiences during and after the COVID-19 pandemic, and their perceived impact on their professional identity and career decisions. Example questions included how students would describe the nursing profession, how the COVID-19 pandemic had changed their lives and influenced their experiences as nursing students, how the pandemic had affected their sense of belonging as both students and future nursing professionals, and how the pandemic had shaped their decision making regarding their education and career choices (Table I). Each interview lasted between 30 and 60 minutes and was audio-recorded with participants’ consent. Field notes were also taken to document nonverbal cues and contextual observations. Participant recruitment continued until the research team determined that data saturation had been reached; that is, no new codes or themes were emerging from subsequent interviews. This approach maximised the depth and diversity of the data while maintaining methodological flexibility to recruit additional participants if new themes continued to emerge. All interviews were conducted in Arabic by one researcher while translated into English by a bilingual member of the research team. The translated interviews were transcribed verbatim for analysis. The translated transcripts were reviewed against the original recordings to ensure accuracy.

**Table I.** Interview Guide Questions.

Questions
**Impact of the COVID-19 Pandemic** 1. How did the COVID-19 pandemic change your life personally? ∘ In what ways did it affect your daily routine, mental health, or family life? 2. How did the COVID-19 pandemic affect your experiences as a nursing student? ∘ Can you describe changes in learning, clinical training, or interactions with faculty and peers? **Sense of Belonging and Professional Identity** 3. How did the COVID-19 pandemic influence your sense of belonging as a nursing student? ∘ Did you feel more connected, disconnected, or unchanged? Why? 4. How do you think the pandemic shaped your identity as a future nursing professional? **Decision-Making and Career Intentions** 5. How did the COVID-19 pandemic influence your decision-making as a nursing student? ∘ Did it affect your confidence, commitment, or future? 6. Did you ever reconsider becoming a nurse during the COVID-19 pandemic? ∘ What led you to question or reaffirm your decision? 7. Do you still want to become a nurse after the COVID-19 pandemic? ∘ Why or why not? 8. Have your motivations or reasons for becoming a nurse changed after the pandemic? ∘ In what ways?

In addition to audiotaped interviews, field notes were systematically maintained throughout data collection. These notes captured immediate observations, contextual details (such as nonverbal cues, setting, and participant demeanour), and interviewer reflections during and after each session. Incorporating field notes provided important context that enriched the interpretation of the audio-recorded data, offering insights sometimes not evident from transcripts alone for example, emotional undertones or shifts in group dynamics. To preserve cultural nuance, translations were reviewed by two independent researchers fluent in both languages, with discrepancies resolved through a consensus-based approach. Reflexive discussions were held to ensure that participants’ meanings were retained as accurately as possible.

### Data analysis

Data were analysed using thematic analysis following the six-phase process outlined by (Braun & Clarke, 2021). This method was selected for its flexibility and suitability in identifying, analysing, and reporting patterns (themes) within qualitative data. The process began with repeated readings of the transcripts to ensure familiarity with the data. Initial codes were then generated inductively from the data, followed by the organisation of the codes into potential themes. These themes were reviewed and refined to ensure internal consistency and accurate representation of the participants’ narratives. With eight participants representing both undergraduate and postgraduate perspectives, many key themes were consistently identified following the interviews. Aligned with qualitative research standards, we considered data saturation as the point when no new codes were seen and thematic sufficiency as the point when adequate data allowed robust theme construction. A reflective approach was maintained throughout the analysis to minimise bias and enhance the interpretive depth. MAXQDA software (version 24) was used for data management and coding.

### Trustworthiness

To ensure the trustworthiness of the study, the criteria outlined by Lincoln and Guba (Lincoln & Guba, 1985), including credibility, dependability, confirmability, and transferability, were applied. Credibility was established through prolonged engagement with the data, peer debriefing, and member checking, during which participants were invited to review the summaries of their interviews for accuracy. Dependability and confirmability were supported by an audit trail that documented the decisions made during data collection and analysis. Transferability was enhanced by providing detailed descriptions of the research context, participant characteristics, and findings, which allowed readers to assess the applicability of the results to other settings. Individual themes were distilled through a rigorous, multi-step thematic analysis process. Verbatim transcripts and accompanying field notes were read repeatedly for immersion, and initial codes were generated manually. Coders compared and discussed coding decisions to refine and group related codes, and themes were constructed by identifying patterns across interviews, drawing upon both spoken content and contextual field observations. This triangulation of data sources, audio, transcription, and field notes, enhanced the richness, transparency, and trustworthiness of our analysis.

### Ethical considerations

Ethical approval was obtained from the Research Ethics Committee (approval number KSU-HE-21-215). All participants received an electronic information sheet outlining the purpose of the study, procedures, confidentiality measures, and rights. Written informed consent was obtained prior to their participation in the interviews from all 8 participants and, each also provided a written informed consent for publication. The Consent forms were signed and returned electronically before scheduling interviews. Participants were assured of the voluntary nature of their participation and their right to withdraw at any time without penalty or consequences. To address potential emotional concerns, interviews were conducted in a supportive and empathetic manner, and information about available support services was prepared, and if needed, provided to participants. No ethical dilemmas or emotional distress were reported by participants during the interviews. The data were anonymized and stored securely on password-protected devices accessible only to the research team. All procedures adhered to ethical guidelines for research involving human participants.

## Results

**Figure 1.**
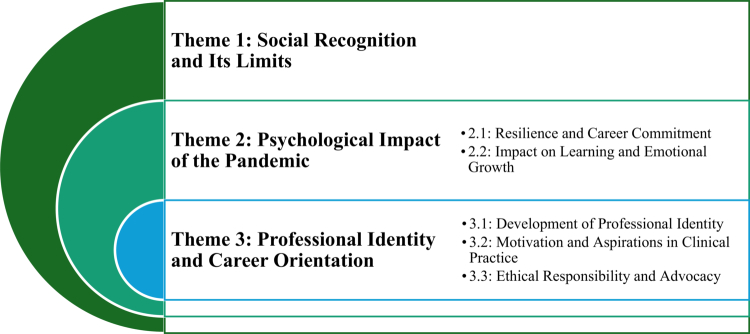
Main themes and subthemes.

Eight nursing students participated in the study, including five BSN students and three MSN students. Most participants were female (*n* = 6) and two male participants. BSN students were in the third or fourth year of their programmes, while MSN participants reported between three and five years of prior clinical experience. Table II summarises the participant characteristics. Three main themes and five subthemes were identified (see Figure 1). Participants shared insights across three interconnected dimensions: social, psychological, and professional. These levels are closely interconnected, often overlapping with participants’ experiences.

**Table II.** Characteristics of participants.

Participant	Gender	Programme	Year of Study/Clinical Experience
*P1*	Female	BSN	Year 3
*P2*	Female	BSN	Year 4
*P3*	Female	MSN	4 years
*P4*	Female	MSN	3 years
*P5*	Female	BSN	Year 4
*P6*	Male	BSN	Year 4
*P7*	Male	MSN	5 years
*P8*	Female	BSN	Year 3

Note: BSN: Bachelor of Science in Nursing; MSN: Master of Science in Nursing

### Main theme 1: social recognition and its limits

This theme explores how the COVID-19 pandemic reshaped societal perceptions of nursing, highlighting both increased public recognition and participants’ perceptions that such appreciation was often temporary or insufficiently translated into sustained professional support. Participants described a shift in societal perceptions, moving from previously held stereotypes and limited recognition toward greater appreciation and visibility of the nursing profession. This external validation was frequently linked to feelings of patriotic responsibility, especially during national health emergencies.

Some students highlighted how public recognition strengthened their sense of pride in the profession. A BSN student noted, “There’s now greater respect and appreciation for nursing” (P5, BSN student), while an MSN student reflected on the heightened visibility of nurses during the pandemic, stating, “All eyes were on us as nurses. It made me proud” (P3, MSN student).

Beyond societal recognition, participants also described nursing as a deeply humanitarian profession rooted in compassion and service. For many, nursing was not merely a career choice but a moral commitment to caring for others. One student explained, “Before it was a field or desire, it was a humanitarian profession. Nursing is more than a specialty; it’s a profession filled with compassion and emotion” (P5, BSN student).

For some students, the pandemic also shaped their developing professional identity. Clinical experiences during this period strengthened their emotional connection to the profession and reinforced their sense of belonging. As one participant stated, “Because I feel like I belong in this profession. I love it, and I’m passionate about it” (P6, BSN student).

However, not all participants expressed sustained optimism. Some noted that the initial societal appreciation during the pandemic gradually faded, while the demands placed on nurses continued to increase. One MSN student expressed this concern: “Society’s support faded once the crisis eased. No matter how much you do, appreciation feels lacking. There’s more pressure, fewer benefits. We deserve respect, training, and fair compensation; not just to be called ‘angels of mercy’” (P7, MSN student).

Overall, participants’ reflections reveal that although the pandemic temporarily enhanced social recognition of nursing, students remained aware of the gap between symbolic appreciation and the need for sustained institutional and societal support for the profession.

### Main theme 2: psychological impact of the pandemic

#### Subtheme 2.1: resilience and career commitment

Several participants described how the pandemic strengthened their professional identity and commitment to nursing. Some students voluntarily assumed high-risk roles, viewing their involvement not as an obligation but as a personal commitment grounded in professional values and moral responsibility. One participant explained, “I volunteered. It was my personal decision… it highlighted the importance of my role. I now feel more committed to the profession” (P7, MSN student).

Despite the challenges posed by the pandemic, participants continued to express strong dedication to clinical practice and professional development. Their resilience was closely tied to a desire to strengthen their practical competencies and remain engaged in direct patient care. As one MSN student explained, “I need to work bedside to improve my skills… Even with a master’s, I want to be hands-on… COVID-19 encouraged me to stick with nursing” (P3, MSN student).

Simultaneously, participants openly acknowledged the emotional toll associated with working during the pandemic, particularly the fear of infecting family members. Both BSN and MSN students described how the responsibility to protect loved ones intensified their emotional burden while reinforcing their sense of professional duty. One MSN student reflected on this concern, stating, “Working in COVID wards caused fear, especially for my family” (P7, MSN student). Similarly, a BSN student described how this fear influenced personal decisions about family contact: “I would have worked with COVID patients if I were a nurse during the pandemic with precautions… I wouldn’t have gone home to avoid infecting my family… I feel like nursing is my place” (P5, BSN student). These narratives illustrate how emotional strain and professional commitment often coexisted during the crisis.

Participants also described how witnessing the pandemic's severity influenced their professional awareness and family decisions. For example, one student emphasised that exposure to critical cases in intensive care units increased his sense of responsibility toward public health, explaining that he encouraged his family to get vaccinated after seeing severe COVID-19 cases in the ICU (P7, MSN student). In addition to fear and stress, some narratives highlighted pride and solidarity within the nursing profession. Students described how observing the dedication of frontline nurses and working alongside colleagues strengthened their sense of belonging to the nursing community. One participant explained that witnessing heroic nursing efforts during vaccination campaigns reinforced his passion for patient care, motivating him to remain in clinical practice rather than pursue an academic or research path (P4, MSN student). Similarly, another student described how seeing the outcomes of collaborative work during clinical shifts increased his motivation and desire to support colleagues during the crisis (P3, MSN student). Together, these reflections illustrate how peer solidarity and collective effort became important psychological sources of motivation during the pandemic.

Overall, participants’ accounts reveal that the pandemic simultaneously generated emotional strain and strengthened professional commitment, highlighting the complex psychological impact of nursing work during a global health crisis.

### Subtheme 2.2: impact on learning and emotional growth

This subtheme describes how the COVID-19 pandemic disrupted the educational experiences of both undergraduate and postgraduate nursing students while also contributing to emotional and professional growth. The sudden transition to online education significantly altered the learning environment and limited opportunities for hands-on clinical training.

Participants described several challenges associated with remote learning, including reduced interaction, limited access to institutional resources, and physical fatigue from prolonged screen use. One student noted that “distance learning was very challenging… I experienced eye strain and headaches” (P4, MSN student), illustrating the physical strain associated with extended online learning. In addition to these physical effects, students reported missing the academic environment and support services available on campus. For example, a BSN student explained, “I missed out on many campus resources like the library, which I loved… it was mostly slides with limited interaction” (P8, BSN student). These experiences suggest that online learning limited both academic engagement and access to institutional learning resources.

Despite these challenges, participants also described how the pandemic encouraged greater independence and personal growth. Many students reported becoming more self-directed in their learning and developing stronger problem-solving skills. For example, one student explained that the pandemic taught them to rely on their own ability to seek and evaluate information independently (P8, BSN student).

Participants further indicated that the pandemic deepened their understanding of the professional responsibilities associated with nursing. Through both clinical exposure and reflective learning, students recognised that nursing requires a combination of technical competence, critical thinking, and interprofessional collaboration. One BSN student explained that effective nursing practice requires “precision, speed, critical thinking, and manual skills acquired through experience and practice” (P1, BSN student). Another participant emphasised the collaborative nature of the profession, describing how nurses work closely with physicians, pharmacists, and other healthcare professionals while maintaining independent clinical judgement (P2, BSN student).

Taken together, these findings indicate that although the pandemic disrupted traditional educational experiences, it also fostered emotional resilience, professional reflection, and a deeper appreciation of the competencies required for effective nursing practice.

### Main theme 3: professional identity and career orientation

#### Subtheme 3.1: development of professional identity

This subtheme illustrates how participants’ professional identities evolved through their educational experiences and clinical exposure during the COVID-19 pandemic. Some participants initially entered the programme with uncertainty or a limited understanding of the profession; however, exposure to clinical environments and reflective learning strengthened their emotional and intellectual connection to nursing. One MSN student described how her perception evolved during training, explaining, “Initially, I had planned to switch, but I grew to love the profession. I now understand and appreciate the nurse’s essential role” (P4, MSN student).

Clinical exposure during training enabled participants to recognise the complexity and professional autonomy embedded in nursing practice. Through hospital placements, participants became more aware of the independent decision-making required in patient care and the profession's multifaceted nature. As one BSN student explained, “When I joined and trained in hospitals, I discovered that nursing involves a lot of independent decision-making” (P1, BSN student).

In addition to developing technical competence, students began to conceptualise nursing as both a scientific and relational discipline that integrates knowledge with empathy and human connection. One participant captured this perspective by describing nursing as “both an art and a science,” where scientific knowledge guides clinical care while empathy and compassion shape patient interactions (P6, BSN student).

Clinical placements played a central role in strengthening professional confidence and communication skills. Experiences in high-acuity settings helped students recognise their own clinical capabilities and reinforced their sense of belonging within the profession. For example, one MSN student noted that performing effectively during intensive care unit training significantly increased his confidence and professional self-assurance (P4, MSN student).

Overall, these findings suggest that clinical exposure, reflective learning, and pandemic-related experiences collectively contributed to the development of a more stable and integrated professional identity among nursing students.

### Subtheme 3.2: motivation and aspirations in clinical practice

This subtheme explores how nursing students envisioned their future professional roles and career trajectories. While many participants expressed a strong commitment to clinical practice, others recognised the diverse career opportunities available within nursing, including education, research, and leadership.

Clinical training often served as an important period of self-discovery, allowing students to identify areas of practice that are aligned with their interests and values. One MSN student explained that her internship experience helped her explore different specialties and ultimately develop a strong connection to paediatric care: “I didn’t have a set plan and used an internship to explore options. In paediatrics, I felt a strong connection to the children I cared for” (P4, MSN student).

Similarly, another participant described how exposure to dedicated frontline nurses influenced her decision to remain in clinical practice rather than pursue an academic path. Observing the dedication and impact of practicing nurses strengthened her motivation to contribute directly to patient care. As she explained, “I initially wanted to work, then go into academia, but now I want to stay in clinical practice. I was inspired by stories of nurses who worked tirelessly and selflessly. I aspire to be like them” (P8, BSN student).

At the same time, participants demonstrated an awareness of the flexibility and diversity of career pathways within nursing. Some recognised that professional fulfilment could also be achieved through roles in research, education, or healthcare administration, reflecting a broader understanding of the profession’s scope. These reflections indicate that students’ career aspirations were shaped by both clinical experiences and evolving perceptions of nursing’s diverse professional opportunities.

### Subtheme 3.3: ethical responsibility and advocacy

This subtheme examines how nursing students articulated a strong ethical orientation toward patient care, public health, and professional advocacy. Participants emphasised that nursing practice involves not only clinical competence but also ethical responsibility toward individuals, communities, and the healthcare system.

Some participants described how their experiences during the pandemic strengthened their awareness of public health responsibilities. For example, one student reflected on the ethical importance of vaccine advocacy and the protection of vulnerable populations, stating, “I felt vaccination was urgent” (P7, MSN student). This perspective illustrates how students viewed nursing ethics as extending beyond bedside care to broaden public health responsibilities.

In addition to public health awareness, several participants expressed aspirations to advocate for the nursing profession and contribute to healthcare leadership. One BSN student explained her ambition to influence healthcare policy and improve professional recognition by pursuing a leadership role within the Ministry of Health: “I want to hold a position in the Ministry of Health, advocate for nurses’ needs, and make nursing more visible” (P2, BSN student).

Ethical responsibility also appeared in students’ reflections on professional accountability and patient safety. Participants emphasised the importance of careful clinical practice, continuous learning, and consultation with experienced colleagues to minimise errors and maintain high standards of care. These perspectives demonstrate students’ internalisation of professional values related to responsibility, integrity, and lifelong learning.

Overall, these findings indicate that students’ professional identities extended beyond clinical competence to encompass ethical responsibility, leadership aspirations, and a commitment to advancing the nursing profession within healthcare systems.

## Discussion

This study examined how the COVID-19 pandemic shaped Saudi undergraduate and postgraduate nursing students’ reflections on professional belonging, career perspectives, and educational experiences during a transitional period following the pandemic's most restrictive phases. The findings suggest that the pandemic created a unique learning and social environment in which students reflected on the meaning of nursing, its societal role, and their potential future within the profession. Although participants reported emotional and academic disruptions, many developed a stronger awareness of the responsibilities and values inherent in nursing practice.

Three overarching themes emerged from the narratives: social recognition and its limits, psychological impact, and professional identity development. Participants described how the pandemic period coincided with increased public attention toward nurses in Saudi Arabia, influencing their perceptions of the profession and their role within it. Heightened visibility often amplified by media coverage and national discourse, contributed to feelings of recognition and social value among some students. Similar observations have been reported in international studies, suggesting that crisis-related visibility of healthcare workers can influence nursing students’ sense of professional belonging and validation (Rosa et al., 2020; Nie et al., 2021). At the same time, several participants noted that their understanding of the profession evolved during their education, particularly as clinical exposure and pandemic-related experiences prompted deeper reflection on the role of nurses within healthcare systems. This interpretation aligns with Tinto (1993) model of student retention, which highlights the importance of academic and social integration in shaping students’ sense of belonging and sustained engagement within professional education programmes.

Levett-Jones and Lathlean (2008) have suggested that a strong sense of belonging is crucial for student engagement, confidence, and retention in nursing education, which are outcomes that were notably reinforced during the pandemic. This highlights how social recognition catalysers the development of professional identity. When students feel acknowledged by society, they internalise a stronger sense of meaning and commitment to nursing. In culturally grounded settings, such as Saudi Arabia, this public admiration translates into national pride, which enhances motivation and reinforces the value of the nursing profession, both within academic spaces and the broader healthcare system. Therefore, public admiration resonated with the students’ sense of national pride and collective duty, reinforcing their professional motivation.

However, these positive shifts occurred alongside significant psychological strains. Participants reported fear of infection, emotional exhaustion, and disruptions to their family life and clinical training. Many displayed resilience and a strong sense of moral responsibility, sometimes even volunteering in high-risk roles, whereas others struggled with feelings of inadequacy or disengagement. This complexity aligns with the findings of Alotaibi et al. (2023), who report that despite concerns about the pandemic, Saudi nursing students generally experienced low levels of fear, anxiety, stress, and obsession, and 86% expressed a positive attitude toward continuing in the nursing profession. These experiences align with Huang et al. (2020), who emphasise that high-pressure environments can cultivate coping skills and moral courage but may also exacerbate vulnerability. Considering (Mezirow, 1991) transformative learning theory, the pandemic served as a disruptive event that triggered self-reflection and redefined professional identity for many, but not necessarily for all. This underscores the importance of viewing resilience not as the absence of difficulty, but as the capacity to persist with purpose amid adversity (Foster et al., 2019).

These findings underscore the importance of integrating emotional preparedness and ethical reasoning into nursing education. Resilience emerges organically for some students; however, it should be nurtured through mental health support, trauma-informed teaching, and crisis-based simulations. Institutions must also consider systemic limitations, including constrained faculty resources, insufficient mental health infrastructure, and unequal access to mentorship, which may hinder these initiatives, particularly in under-resourced academic settings.

The pandemic has also triggered a marked shift in students’ career orientations. Some initially expressed doubts about remaining in nursing or considered switching to other fields, such as quality management or academia. Nevertheless, real-world exposure during internships helped reframe nursing as a multifaceted discipline. This view aligns with Benner et al. (2009), who emphasise the experiential development of professional identity. Students voiced their aspirations for public health, research, health informatics, and policy, reflecting a shift from narrow clinical roles to broader system-level engagement. Most participants expressed a desire for ethical leadership, inclusion, and systemic equity, highlighting a maturing awareness of the structural challenges in healthcare. Brolan et al. (2022) emphasise the importance of feeling recognised by leadership, which positively impacts morale and engagement. These findings align with calls for nursing curricula to incorporate advocacy, leadership development, and political literacy (Udod, 2023).

The sense of belonging described by participants during the pandemic may represent an important moment in students’ professional identity development. Rather than indicating a temporary response to crisis conditions, these reflections suggest that experiences during the pandemic shaped students' understanding of the meaning, responsibilities, and societal role of the nursing profession. These findings suggest that crisis contexts can shape students’ professional perspectives and encourage deeper reflection on their future roles in healthcare. The challenge lies in institutionalising a sense of inclusion. These findings highlight the potential importance of cultivating psychologically safe and inclusive learning environments, supported by mentorship and collaborative learning opportunities (Kelly et al., 2024; Singer et al., 2022, 2024). Reflection tools, such as journaling and debriefing, can support identity development, emotional regulation, and long-term engagement.

The shift toward hybrid learning has also introduced a range of complex challenges. Students entering nursing programmes during periods of remote learning or clinical disruption may experience fragmented identity development. Lin et al. (2024) confirm that limited real-world engagement can delay students’ integration into professional roles. Therefore, creating intentional structures that promote inclusion and engagement across digital and physical learning spaces is essential.

Maslow (1943) hierarchy of needs offers a valuable lens to understand the multi-layered impact of the pandemic on nursing students’ sense of belonging, resilience, and professional identity development. The pandemic threatened students’ basic physiological safety through fear of infection and disruption of clinical training and challenged psychological and social needs related to belonging and esteem. Our findings reveal that when students’ belonging needs were met through increased social recognition, institutional support, and peer connections, their motivation and commitment to nursing strengthened, aligning with Maslow (1943) notion that belongingness is a critical prerequisite for higher-order needs such as self-esteem and self-actualisation. Thus, nursing education must consider strategies that holistically address students’ needs, from ensuring safe learning environments to fostering social inclusion and professional growth opportunities.

While this study provides valuable insights, its scope was limited to specific demographic and institutional contexts. Future research should explore broader populations, including private and rural institutions, as well as comparative international cohorts. Moreover, future studies could examine voices underrepresented in this study, such as those who experienced alienation or disengagement during the pandemic, to provide a more nuanced and inclusive understanding.

### Limitations

This qualitative study adhered to the Standards for Reporting in Qualitative Research (SRQR) (O’Brien et al., 2014). Nonetheless, this study had several limitations. Primarily, the findings reflect the experiences of students who consented to participate; those who chose not to participate may have had different perspectives.

An additional limitation relates to the timing of data collection. The interviews were conducted after the most restrictive phases of the COVID-19 pandemic but before longer-term professional trajectories could be observed. Consequently, participants’ reflections capture experiences during a transitional period rather than offering insight into the pandemic's longer-term impact on students’ professional development after graduation or entry into the workforce.

The sample comprised both undergraduate students in a pre-licensure BSN programme and postgraduate students in an MSN programme, introducing variation in academic level and prior clinical experience. However, no formal data were collected regarding participants’ years of professional experience or previous nursing roles, which could have influenced perspectives particularly among postgraduate students who may have practiced as nurses before enrolment. The inclusion of both groups adds diversity and depth; however, the small sample size and absence of detailed experience stratification limit the transferability of the findings. These issues could be explored in future studies involving broader and more diverse student populations.

Nonetheless, this study prioritised transparency by providing a detailed account of the research process, as transferability is not the primary aim of qualitative research design. Future research should consider explicitly examining the influence of prior professional experience and work readiness on students’ sense of belonging and educational engagement. Additionally, as this study’s sample was limited to three universities, it would be beneficial for future researchers to broaden its scope by collecting data from a larger number of institutions, including both public and private universities. Interviews were conducted in Arabic to allow participants to express themselves comfortably; however, transcription, translation into English, and cross-verification against audio recordings proved challenging and time-consuming.

As qualitative researchers, we acknowledge that our professional background and cultural context have shaped our interpretations. Reflexive discussions among the research team helped mitigate individual biases and ensured a balanced representation of the participants’ voices. Nevertheless, future work would benefit from participatory or co-interpretive methods to further centre student perspectives on the meaning-making process.

#### Implications and recommendations

To strengthen nursing students’ sense of belonging, classroom educators should cultivate inclusive environments through active dialogue, promote equitable engagement, and facilitate social connections among students. Encouraging open dialogue, affirming diverse student identities, and counteracting implicit bias are essential to fostering belonging in the academic setting. Clinical placement educators play a crucial role by warmly welcoming students, integrating them into healthcare teams, offering supportive mentorship, and recognising their contributions. Both cohorts can collaborate to ensure consistent messaging about professional values and support networks.

Several key strategies are recommended to sustain nursing students’ sense of belonging, particularly in the evolving post-pandemic context. First, establishing structured mentorship programmes can provide students with consistent guidance and professional socialisation by pairing them with clinical or academic role models (Kelly et al., 2024; Singer et al., 2024). Second, integrating reflective practices such as journaling and debriefing in both classroom and clinical environments promotes emotional processing, critical thinking, and a deeper connection to professional identity (Foster et al., 2019). Third, inter-professional learning experiences foster collaboration and shared purpose, enhancing inclusion within broader healthcare teams (Ulenaers et al., 2021). Fourth, promoting resilience through psychological self-care, supportive systems, and healthy work environments is vital to maintaining well-being and effectiveness (Cooper et al., 2020; Brolan et al., 2022). Finally, encouraging leadership involvement from student councils to curriculum feedback and quality improvement initiative strengthens students’ sense of agency and institutional belonging, supporting motivation and retention (Singer et al., 2024; Udod, 2023).

Several participants expressed aspirations beyond bedside care, including leadership, research, and health policy roles, reflecting a broader career vision shaped by pandemic experiences. This underscores the importance of institutional advocacy such as mental health services, ethical support forums, and policy reform to support nurses presently and in future crises (Rosa et al., 2020). Participants’ ethical awareness underscores the need for curricula that foster political literacy and understanding of health policy, preparing nurses to lead within complex healthcare systems.

Suggestions for future research include evaluating targeted interventions by classroom and clinical educators to enhance belonging, investigating cohort-specific experiences of belonging, and studying the long-term impacts of belonging on academic and professional trajectories. These focused studies can generate actionable insights tailored to academic and clinical education contexts.

## Conclusion

This study highlights how the COVID-19 pandemic context shaped Saudi nursing students’ reflections on professional identity, belonging, and their educational experiences. The findings suggest that periods of crisis can prompt students to reconsider the meaning and responsibilities of the nursing profession while strengthening awareness of its societal role. Supporting students’ sense of belonging through inclusive learning environments, structured mentorship, reflective practices, and opportunities for collaboration may therefore play an important role in sustaining engagement in nursing education.

These findings also highlight the importance of preparing nursing students not only for clinical practice but also for broader professional roles, including leadership, research, and advocacy within healthcare systems. Future research should explore these dynamics across diverse educational contexts and follow students into their early professional careers to better understand how crisis-related experiences influence professional identity development over time.

## Data Availability

Upon request, data will be made available. The data that support the findings of this study are available from the corresponding author, MB, upon reasonable request.
